# Lipid Droplets in Cancer: Guardians of Fat in a Stressful World

**DOI:** 10.3390/molecules23081941

**Published:** 2018-08-03

**Authors:** Toni Petan, Eva Jarc, Maida Jusović

**Affiliations:** 1 Department of Molecular and Biomedical Sciences, Jožef Stefan Institute, Ljubljana SI-1000, Slovenia; eva.jarc@ijs.si (E.J.); maida.jusovic@gmail.com (M.J.); 2 Jožef Stefan International Postgraduate School, Ljubljana SI-1000, Slovenia

**Keywords:** lipid droplets, fatty acids, autophagy, lipophagy, cancer, oxidative stress, nutrient stress, lipotoxicity, lipase, phospholipase

## Abstract

Cancer cells possess remarkable abilities to adapt to adverse environmental conditions. Their survival during severe nutrient and oxidative stress depends on their capacity to acquire extracellular lipids and the plasticity of their mechanisms for intracellular lipid synthesis, mobilisation, and recycling. Lipid droplets, cytosolic fat storage organelles present in most cells from yeast to men, are emerging as major regulators of lipid metabolism, trafficking, and signalling in various cells and tissues exposed to stress. Their biogenesis is induced by nutrient and oxidative stress and they accumulate in various cancers. Lipid droplets act as switches that coordinate lipid trafficking and consumption for different purposes in the cell, such as energy production, protection against oxidative stress or membrane biogenesis during rapid cell growth. They sequester toxic lipids, such as fatty acids, cholesterol and ceramides, thereby preventing lipotoxic cell damage and engage in a complex relationship with autophagy. Here, we focus on the emerging mechanisms of stress-induced lipid droplet biogenesis; their roles during nutrient, lipotoxic, and oxidative stress; and the relationship between lipid droplets and autophagy. The recently discovered principles of lipid droplet biology can improve our understanding of the mechanisms that govern cancer cell adaptability and resilience to stress.

## 1. Introduction

Cancer cells are often faced with an inconsistent and limited availability of nutrients due to poor vascularization [1]. They possess powerful oncogene-driven mechanisms of metabolic reprogramming that enable cell survival and growth even in the harshest environmental conditions [2,3]. Their remarkable adaptability to adverse conditions is critical for their propensity to migrate and invade neighbouring and distant tissues [1]. Cancer cells exposed to severe stress display an impaired ability to synthesize their own fatty acids (FAs) and their survival depends on lipids from the microenvironment and on those that can be recommissioned from their own structural and storage pools [3,4]. Cancer cells having greater capabilities to acquire extracellular lipids and efficient mechanisms for intracellular lipid mobilisation and recycling will thus have a significantly higher chance for survival. Indeed, some of the most aggressive cancer types driven by the Ras-oncogene use opportunistic modes of FA acquisition, possess an increased capacity for FA accumulation in intracellular lipid droplets, and upregulate mitochondrial FA oxidation to resist nutrient and oxidative stress [5,6,7,8,9,10]. Lipid droplets are dynamic organelles that play a pivotal role in the management of lipid uptake, distribution and consumption. Lipid droplet accumulation has been observed in many cancers and is elevated in cancer cells exposed to hypoxia or nutrient starvation [8,11,12,13,14,15]. Emerging studies suggest that these organelles suppress nutrient and oxidative stress and contribute to cancer cell survival and growth [7,8,9,15,16,17,18,19], suggesting that lipid droplet metabolism is an attractive target for reducing the resilience of cancer cells to stress.

**Figure 1:**
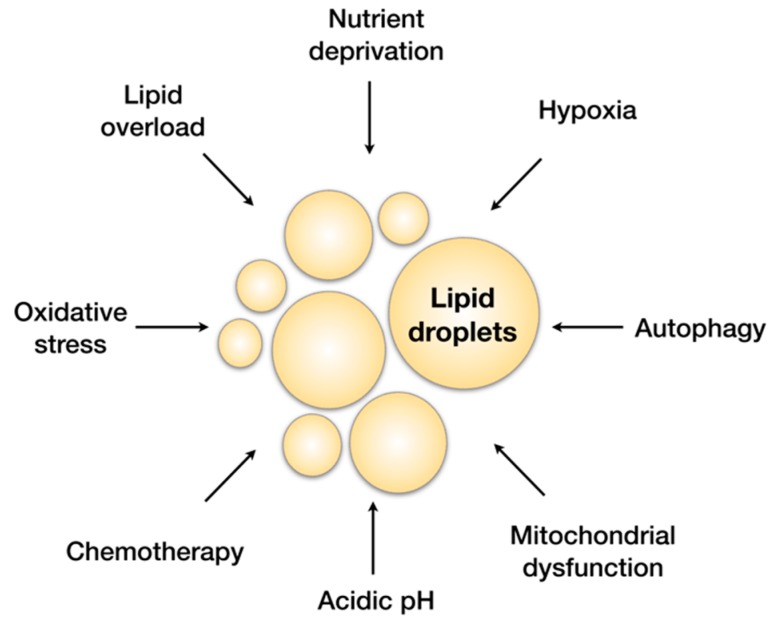
Lipid droplet biogenesis is induced during stress. Environmental factors and cellular states that are characterised by imbalances in cellular energy metabolism and redox homeostasis, and are typical of tumour-like stress, induce the biogenesis of lipid droplets, including lipid overload, complete or partial nutrient and oxygen deprivation, oxidative stress, high autophagic flux, acidic environment, mitochondrial damage and apoptotic cell death, and treatment with chemotherapeutics.

**Figure 2:**
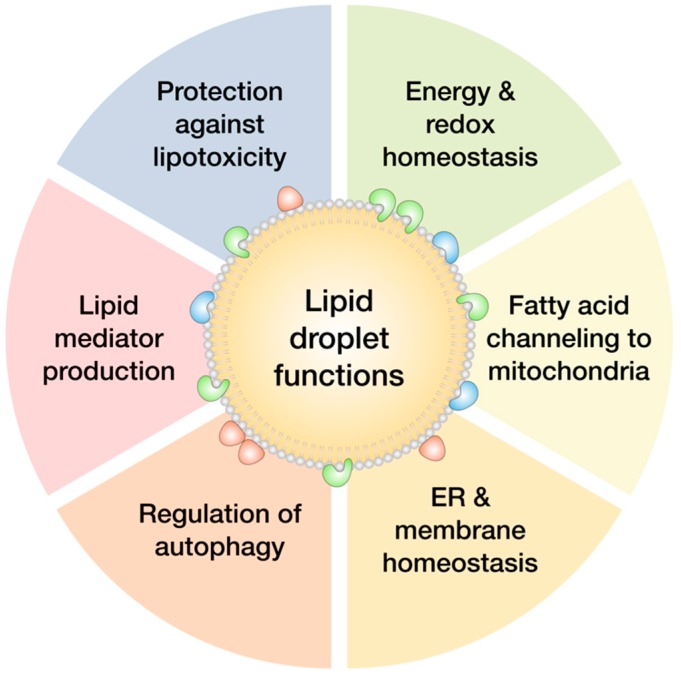
Lipid droplet functions during stress. Lipid droplets can assume different roles in stressed cells: (1) reduction of lipotoxic cell damage by sequestration of potentially toxic endogenously- or exogenously-derived lipids, such as saturated and polyunsaturated fatty acids (FAs), ceramides and cholesterol, and storing them in the form of inert triacylglycerols (TAGs), acylceramides, and cholesterol esters; (2) maintenance of energy and redox homeostasis by providing substrates for energy production, stimulating NADPH synthesis, and regulating oxidative metabolism via lipolysis-mediated signalling; (3) regulation of FA trafficking and distribution, e.g., enabling optimal transfer of FAs to mitochondria for efficient FA oxidation; (4) maintenance of endoplasmic reticulum (ER) and membrane homeostasis, protein quality control, and protection against ER stress; (5) regulation of autophagy; and (6) production of bioactive lipid mediators, including pro- and anti-inflammatory signalling molecules derived from polyunsaturated FAs, such as eicosanoids and specialized pro-resolving mediators.

Lipid droplets store neutral lipids, mostly triacylglycerol (TAG) and sterol esters, and balance lipid uptake, storage and use according to cellular needs [20]. They have long been regarded as inert fat depots, but numerous roles beyond energy storage are emerging, including regulation of lipid trafficking, protein management and quality control, and viral replication [18,19,21,22]. Most cells respond to lipid overload by synthesizing lipid droplets to dampen the potentially damaging surge of lipids, but, intriguingly, their biogenesis is also induced during various types of stress, including complete deprivation of nutrients and lipids (Figure 1). It is becoming clear that a common feature of the cellular states that promote lipid droplet production are imbalances in energy metabolism and redox homeostasis. These may be instigated by different stressors, such as complete or partial nutrient deprivation, nutrient overload, oxygen deprivation, treatments with pro-oxidants, ceramides, chemotherapeutic agents, and inducers of endoplasmic reticulum (ER) stress [7,8,9,16,23,24,25,26,27] (Figure 1). These conditions lead to the activation of stress-response pathways and result in different cellular defects that have been shown to induce lipid droplet biogenesis: oxidative stress [8,24,26,28,29], ER stress [30], activation of autophagy [31,32,33,34], inflammation [13,35], mitochondrial dysfunction, and cell death [36,37,38]. The fact that cells undergo a seemingly futile cycle of lipid droplet biogenesis and breakdown during such challenging conditions suggests that the benefits of lipid droplets must outweigh the expense of their biogenesis. Indeed, recent studies reveal the pleiotropic nature of lipid droplets and their broad impact on cellular homeostasis during stress. It is now clear that some of the major roles of lipid droplets are associated with the management of cellular stress (Figure 2). 

First, lipid droplets are central anti-lipotoxic organelles that control FA, diacylglycerol (DAG), cholesterol and ceramide lipotoxicity by sequestering these potentially toxic lipids into inert triglycerides, cholesterol esters and acylceramides [9,27,39,40,41] (see Section 4 and Section 5). The biogenesis of lipid droplets is protective because it reduces direct cell damage caused by these lipids and reduces their availability for conversion into other toxic lipid species. Importantly, recent studies have shown that lipid droplets not only protect cells from exogenous lipid overload, but that they also guard against a surge of endogenous lipids, such as that occurring during high autophagic flux [31,32]. Lipid droplets also sequester oxidation-prone polyunsaturated FAs in their protective core, thus restricting lipid peroxidation reactions and maintaining optimal membrane saturation and organelle homeostasis during hypoxia, oxidative stress, lipid overload, and apoptotic cell death [9,24,37]. Lipid droplets are thus crucial for managing lipid imbalances arising due to different stress conditions and are critical for diverting potentially toxic lipids from a bioactive or a structural pool to a storage pool within the protective neutral lipid droplet core. 

Second, lipid droplets provide FAs not only for energy production during both nutrient and oxygen deprivation, but also for the maintenance of redox homeostasis (see Section 6 and Section 7). In cancer cells exposed to hypoxia, FA uptake is upregulated and lipid droplets are formed to protect cells from reactive oxygen species (ROS) by supporting NADPH synthesis [8]. They also provide temporary storage for FAs that may be used as needed during the rapidly changing conditions in the microenvironment, such as hypoxia/reoxygenation and feeding/starvation [7,8,9,17]. Lipid droplets thus act as switches that coordinate FA trafficking and consumption either as fuel for energy production, for the maintenance of redox homeostasis or for membrane biogenesis during rapid cell growth [19,42,43]. 

Third, lipid droplets dynamically interact with mitochondria in response to nutrient cues and provide a network of conduits for efficient channelling of FAs, thereby enabling a homogenous distribution of FAs into tubulated mitochondria and maximizing the efficiency of FA oxidation during starvation (see Section 6) [25,41]. However, recent studies have only begun to reveal the complexity of the relationship between mitochondria and lipid droplets. Intriguingly, a subset of peridroplet mitochondria with an elevated TCA cycle capacity but a reduced FA oxidation, in fact, provides ATP for the synthesis of TAG and supports lipid droplet biogenesis [44].

Fourth, lipid droplets are crucial for the maintenance of membrane and ER homeostasis. They contribute to membrane and protein quality control and are involved in the removal of damaged lipids, aggregated and unfolded proteins, thereby reducing ER stress [16,19,26,40,45,46,47]. Emerging studies suggest that alterations in lipid droplet breakdown through lipolysis and autophagy have a profound effect on the management of membrane and ER homeostasis, oxidative stress, mitochondrial function, and stress signalling (see Section 5, Section 7 and Section 8) [48,49,50,51,52,53,54,55,56,57]. The breakdown of lipid droplets leads to the release of hundreds of lipid species with different characteristics and cellular roles, including FAs, DAGs, ceramides, and cholesterol. An excessive lipid droplet turnover may cause cell damage, and the balance between lipid droplet biogenesis and breakdown must be tightly controlled by cells exposed to stress. 

Fifth, lipid droplets are engaged in a complex relationship with autophagy (see Section 8 and Figure 5). On the one hand, lipid droplets have been shown to provide lipids for the formation of autophagosomal membranes, manage ER stress, or stimulate signalling processes that promote autophagy [58,59,60,61]. On the other hand, autophagy drives lipid droplet biogenesis by providing lipids recycled from membranous organelles [25,31,34], but it may also participate in their breakdown through lipophagy, a lipid droplet-selective form of autophagy [62].

Finally, the breakdown of lipid droplets provides FAs and other lipids that act as signalling molecules by themselves—e.g., by interacting with transcription factors such as peroxisome proliferator-activated receptors (PPARs) and sterol-regulatory element binding proteins (SREBPs)—or they are converted into bioactive lipid mediators, such as eicosanoids, that act as paracrine and autocrine messengers affecting inflammatory signalling, metabolism, proliferation, migration, and metastasis [14,63,64,65,66]. This emerging aspect of lipid droplet biology will not be covered in this review, but we would like to refer the reader to several excellent recent reviews [13,18,67,68,69].

In this review, we focus on recent advances describing the involvement of lipid droplets in the protection against nutrient, lipotoxic, and oxidative stresses. We discuss the emerging mechanisms of stress-induced lipid droplet biogenesis, the roles of lipid droplets during stress, and the complex bidirectional relationship between lipid droplets and autophagy. Because studies on the role of lipid droplets in cancer are relatively scarce, we provide a broader overview of lipid droplet function in cellular stress in various tissues and pathophysiological settings. Many of these recently discovered principles of lipid droplet biology can improve our understanding of the mechanisms that govern cancer cell resistance to stress. 

## 2. Lipid Droplets Basics 

Lipid droplets have a unique structure among organelles with a central core of neutral lipids surrounded by a phospholipid monolayer and numerous peripheral and embedded proteins [70,71,72]. Their neutral lipid core is composed mostly of TAGs and sterol esters, but they also store acylceramides and fat-soluble vitamins [18,39]. These are cytosolic organelles, derived from the ER, but have also been observed in the nucleus and may be produced at the inner nuclear membrane in yeast cells [73,74]. The most widely accepted mechanism of lipid droplet biogenesis posits that lipid droplets are formed de novo following the synthesis of TAGs and sterol esters between the two leaflets of the ER membrane [70,75,76]. Diacylglycerol acyltransferases 1 and 2 (DGAT1 and DGAT2) catalyse the last step in the TAG biosynthesis pathway, while cholesterol ester (CE) synthesis depends on acyl-CoA cholesterol acyltransferases 1 and 2 (ACAT1 and ACAT2). When sufficient lipids accumulate within the growing lipid lens in the ER bilayer, a nascent lipid droplet buds from the ER membrane and is released into the cytosol but still maintains dynamic membrane contacts with the ER, which enable bidirectional cargo transfer [77,78]. Many components of the lipid droplet biogenesis machinery are transferred to the nascent lipid droplet and enable the growth, fusion, and fission of these dynamic organelles. Lipid droplets undergo a dynamic cycle of biogenesis and breakdown in response to nutrient cues from the environment. They differ in size, number and distribution within cells in a population and in different cell types and these properties change depending on the physiological state of the cell [79]. Different lipid droplet pools are thought to have different protein compositions and functions [71,80,81,82]. 

Lipid droplets are broken down by two major mechanisms: lipolysis and lipophagy [51,57,83,84]. Lipolysis enables a highly regulated release of FAs from TAGs by the sequential action of adipose triglyceride lipase (ATGL), hormone-sensitive lipase (HSL), and monoacylglycerol lipase (MAGL) [85,86,87]. Lipid droplet breakdown in adipocytes is hormonally regulated and provides FAs for mitochondrial energy production in non-adipose tissues during fasting [66,85,86]. In non-adipose tissues, lipolysis is involved in the regulation of mitochondrial oxidative metabolism, FA oxidation, membrane synthesis, cell growth, ER homeostasis, and lipid mediator synthesis [25,40,43,63,66,88,89,90]. Lipophagy is a recently discovered selective form of autophagy, whereby lipid droplets are enclosed within autophagosomal membranes and fused with lysosomes for degradation by hydrolytic enzymes [62]. Neutral and lysosomal lipolysis have overlapping roles in cellular lipid degradation and a cross-talk between them has been proposed [51,56,91]. This and other aspects of lipid droplet turnover in cells exposed to stress is discussed in more detail in the following sections. 

## 3. Cancer Cells Depend on Lipids to Survive and Grow in a Challenging Environment 

**Figure 3:**
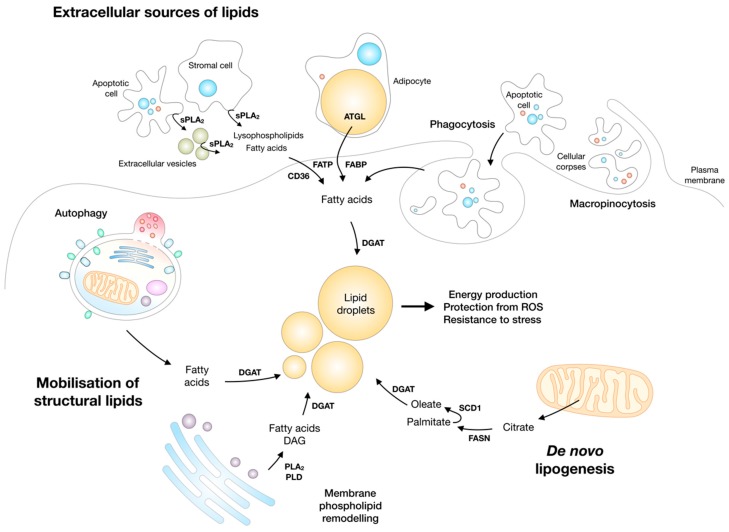
Different pathways of lipid acquisition may drive lipid droplet biogenesis in stressed cancer cells. Depending on the level of nutrient and oxygen deprivation and on the availability of extracellular lipids, cells may interchangeably use three main lipid pools: (1) extracellular sources, particularly when access to circulating lipids is limited, cancer cells display alternative and opportunistic modes of lipid uptake and may acquire fatty acids (FAs) from extracellular lysophospholipids, from stromal adipocytes through adipose triglyceride lipase (ATGL)-mediated lipolysis, via membrane phospholipid hydrolysis of neighbouring cells, extracellular vesicles and apoptotic cells by enzymes such as secreted phospholipase A_2_ (sPLA_2_), and by engulfing various material, such as apoptotic cells and even cellular corpses; (2) endogenous de novo FA synthesis, which becomes dominant when extracellular lipid sources are exhausted; and (3) mobilisation of endogenous, structural lipids via breakdown of membranous organelles by autophagy and/or membrane phospholipid hydrolysis by phospholipases A_2_ (PLA_2_) and D (PLD), which is activated during severe stress, such as complete nutrient deprivation. CD36, cluster of differentiation 36, platelet glycoprotein 4, fatty acid translocase; DAG, diacylglycerol; DGAT, diacylglycerol acyltransferase; FABP, fatty acid-binding protein; FASN, fatty acid synthase; FATP, fatty acid transport protein; SCD1, stearoyl-CoA desaturase 1.

**Figure 4:**
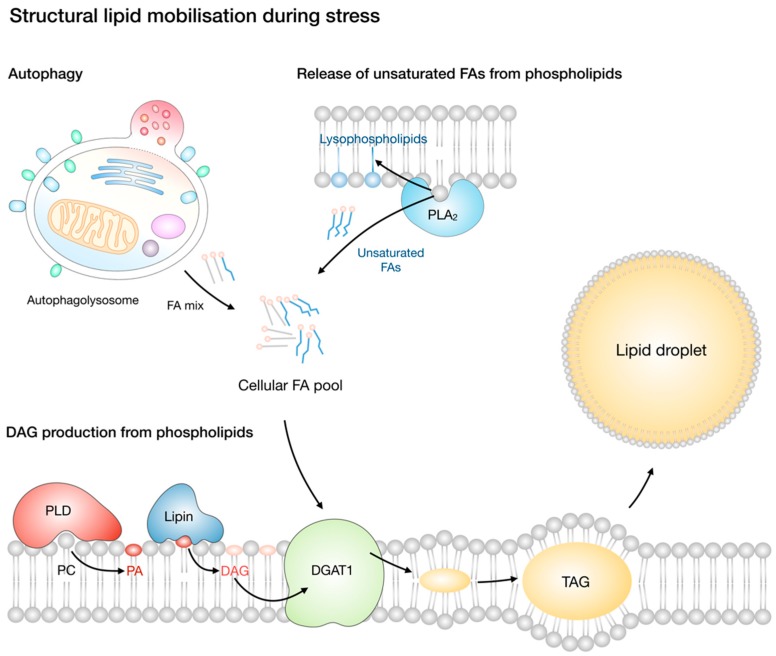
Autophagy and phospholipase-mediated phospholipid hydrolysis stimulate lipid droplet biogenesis during stress. Nutrient starvation activates the autophagy-mediated breakdown of membranous organelles leading to the release of various saturated and unsaturated fatty acids (FAs) that drive diacylglycerol acyltransferase 1 (DGAT1)-mediated lipid droplet biogenesis. Cellular stress may also lead to phospholipase A_2_ (PLA_2_) activation and membrane phospholipid hydrolysis that predominantly results in the release of unsaturated and polyunsaturated fatty acids (FAs), which are typically found at the *sn*-2 position of membrane phospholipids. Intracellular phospholipases A_2_, such as the calcium-independent group VIA PLA_2_ (iPLA_2_β or PNPLA9), or secreted isoforms, such as the group X secreted PLA_2_, may drive lipid droplet biogenesis. Oxidative stress may lead to phospholipase D (PLD)-mediated redistribution of FAs from membrane phospholipids to triacylglycerols (TAGs) stored within lipid droplets in order to protect polyunsaturated FAs from peroxidation. Phospholipase D (PLD) converts phosphatidylcholine (PC) to phosphatidic acid (PA), whereas phosphatidate phosphatases (lipins) convert PA to diacylglycerol (DAG). The latter is then available for TAG synthesis by DGAT1 or DGAT2. Phospholipase activity-derived FAs could be preferred when a specific phospholipid and/or TAG acyl-chain remodelling is required instead of the bulk FA mixture provided by autophagy. Future studies will provide clues on the possible crosstalk between these pathways and the conditions that govern the predominant mechanisms of lipid droplet biogenesis during various conditions of stress in different cell types.

During tumorigenesis, cancer cells acquire various metabolic alterations to overcome the metabolic challenges of rapid proliferation and survival under inhospitable conditions. The changes in cancer metabolism are driven by oncogene activation or loss of tumour-suppressor genes, and by alterations in cellular signalling [2,92,93]. Recent evidence has revealed that the reprogramming of lipid metabolism, including changes in de novo lipogenesis, FA oxidation, as well as phospholipid and neutral lipid metabolism, is essential for various aspects of tumourigenesis [12,94,95,96,97,98]. While most normal cells preferentially use extracellular lipids for the synthesis of new structural lipids, cancer cells can elevate de novo FA synthesis irrespective of the levels of extracellular lipids to satisfy their requirements for lipids or to sustain proliferation in a lipid-poor microenvironment [94,98]. Upregulated SREBP signalling contributes to increased phospholipid, TAG, and cholesterol syntheses, and promotes cell survival and tumour growth [99,100]. On the other hand, FA oxidation is essential for the survival of cancer cells undergoing metabolic stress due to starvation, hypoxia, and matrix-detachment [95,101]. Furthermore, cancer cells display alterations in nutrient acquisition pathways and are able to scavenge extracellular lipids to sustain proliferation even in nutrient- and oxygen-deprived conditions [3,4,5,8,10]. In times of need, cancer cells use opportunistic modes of nutrient acquisition [2] and extract lipids from unusual sources, such as lysophospholipids [5] and even necrotic cellular corpses [10]. Cancer cells also engage in symbiotic relationships with neighbouring adipocytes to obtain FAs for energy production [102,103,104]. Numerous lipids, such as sphingolipids, FAs, and their oxygenated metabolites eicosanoids and pro-resolving lipid mediators, act as signalling molecules and second messengers that alter cancer cell proliferation, migration, angiogenesis, and immune responses [69,94,105]. Finally, membrane lipid composition, levels of saturation and cellular distribution of lipids are underexplored aspects that are crucial for organelle homeostasis, cell signalling, and the management of nutrient and oxidative stress [9,94,106,107]. It is becoming clear that the regulation of lipid metabolism, and in particular FA availability, is critical for the survival and proliferation of the stressed cancer cell. It is thus not surprising that cancer cells have multiple sources of FAs at hand that may be interchangeably used according to their particular metabolic needs and nutrient availability. Cancer cells exposed to limiting levels of nutrients and oxygen may acquire FAs from at least three main lipid pools (Figure 3), which may all drive lipid droplet biogenesis: (1) extracellular sources, including unusual, “alternative” sources of lipids and opportunistic modes of acquisition that provide FAs from extracellular lysophospholipids [5], from neighbouring adipocytes through ATGL-mediated lipolysis [102,103], through membrane phospholipid hydrolysis of healthy or damaged stromal cells and extracellular vesicles by enzymes such as secreted phospholipases A_2_ (sPLA_2_s) [7,108,109,110,111] and by engulfing damaged cells and cell remnants, such as apoptotic cells and cellular corpses [2,10]; (2) de novo FA synthesis, which is significantly upregulated in several cancers and is activated in the absence of extracellular lipids [94]; and (3) mobilisation of endogenous, structural lipids via breakdown of membranous organelles by autophagy and/or membrane remodelling by phospholipases (see Section 6 and Section 8.3 and Figure 4) [7,9,15,23,24,108,112]. Lipid droplets are emerging as consolidating and regulatory hubs for these pathways of cellular lipid acquisition and are thus attractive targets for the management of cellular stress in cancer.

### Lipid Droplets Accumulate in Various Cancers

Some of the earliest evidence for the existence of cytoplasmic lipid droplets in cancer cells was provided in 1957 when numerous lipid droplets were found in the cytoplasm of a rat liver tumour [113]. The first lipid droplets in human cancer cells were observed in 1963 when a high content of stainable “lipid vesicles” was observed in mammary carcinoma [114]. In a breakthrough study, Ramos and Taylor [115] performed a morphologic characterization of tumours from clinical samples and found that lipid-containing mammary carcinomas display a more aggressive behaviour. Since then, lipid droplets have been described in various animal and human cancers [116,117,118] and their accumulation has been associated with neoplastic processes and tumour invasiveness. Lipid droplet accumulation has been observed in many human cancers including breast [114,119], brain [118], liver [116], lung [120], cervical [121], prostate [122], colon [14], skin, bile duct [123], clear-cell renal cell carcinoma [124], ovarian [103], and pancreatic cancers [125]. A high aggressive potential has been ascribed to lipid-rich tumours and it has been associated with a poor clinical outcome [125,126]. Elevated lipid droplet accumulation has also been observed in hypoxic tumour areas and cancer cell lines deprived of oxygen [8,29,99,127]. It has been suggested that elevated lipid droplet levels in tumours are a consequence of acidic pH, glucose deprivation, or hypoxia [11]. These days, it is clear that cancer cells display significant alterations in lipid metabolism [94] and emerging evidence suggests that changes in lipid droplet metabolism are an important hallmark of cancer metabolic reprogramming [12,13,92,128]. 

## 4. Lipid Droplet Biogenesis and Lipotoxicity 

Cells maintain FA homeostasis by coordinating the various cellular processes that generate or deliver FAs with processes that use these molecules. They have to balance their endogenous FA levels with FA availability from the circulation and according to their needs for energy production in mitochondria, for use as building blocks for membranes, and as precursors for more complex signalling lipids. The main sources of exogenous lipids in the circulation are serum albumin-FA complexes, released by adipocytes during fasting/lipolysis and lipoproteins, providing FAs released by lipoprotein lipase or other lipases/phospholipases [57,129]. The major sources of cellular endogenous FAs are de novo FA synthesis and TAG lipolysis from existing lipid droplets, but mobilisation of structural lipids by autophagy of membranous organelles and by membrane phospholipid hydrolysis also contributes to the endogenous pool of FAs, particularly during nutrient deprivation [7,15,23,25,112] (discussed in more detail in Section 6 and Section 8.3; Figures 3 and 4). Any imbalance in these processes may lead to excess amounts of free FAs in the cell, which leads to lipotoxicity. Lipotoxicity is thus most often a consequence of a disparity between cellular lipid uptake, storage, and utilization [130]. Cells exposed to increased levels of FAs experience cellular stress, dysfunction and damage associated with elevated lipid turnover, ROS production, ER stress, mitochondrial damage, defective cell signalling pathways, impaired insulin signalling, elevated production of inflammatory mediators and cell death [130,131,132]. Lipid overload increases the rates of processes associated with FAs, such as β-oxidation, which may consequently lead to the over-production of ROS, lipid peroxidation, membrane, DNA and protein damage [133]. It is therefore not surprising that chronically elevated circulating levels of lipids and ectopic lipid accumulation in tissues have been linked with debilitating chronic diseases such as obesity, metabolic syndrome, heart failure, liver diseases, and diabetes [21,130,133]. In addition, besides diabetes and liver diseases, obesity is now a well-recognized risk factor for the development of many cancer types and it has been associated with reduced patient responses to chemotherapy [134,135].

As discussed in the following paragraphs in more detail, accumulation of excess FAs in the protective lipid droplet core diverts FAs from pathways causing lipotoxicity, thereby reducing the biogenesis of lipid species with a greater lipotoxic and pro-inflammatory potential, such as DAGs, ceramides, and acylcarnitines [31,39,136,137,138], and protecting sensitive, oxidation-prone polyunsaturated FAs from lipid peroxidation [9,24]. Lipid droplets also suppress nutrient stress associated with lipid overload by reducing excess mitochondrial FA oxidation and ROS overflow [132,139]. Indeed, mechanisms that control lipid droplet breakdown are critical for balancing FA-mediated energy production, FA trafficking, and oxidative metabolism. A poorly explored factor important for lipotoxicity is the “lipid quality” within lipid droplets, i.e., the FA composition of TAGs and CEs stored in lipid droplets. The acyl-chain composition of lipid droplets, together with enzymes regulating lipid droplet synthesis and breakdown, governs the availability of particular FA species for various cellular processes, including the regulation of membrane unsaturation, which is tightly connected with ER and redox homeostasis during stress. Lipid droplets thus generally serve as protective organelles, which is seemingly at odds with the fact that lipid accumulation in non-adipose tissues has been associated with impaired cellular function and metabolic diseases. This paradox likely occurs due to the fact that in pathologic states, lipotoxicity may occur over time when the cellular capacity for neutral lipids is exceeded leading to dysregulation of lipid droplet biogenesis and/or breakdown mechanisms. In such conditions, lipolysis may be chronically elevated leading to cell damage associated with increased free FA levels in the cytosol [55,130,132]. 

### 4.1. Lipid Droplet Biogenesis Protects from Lipotoxicity

Excess FAs may be either removed by oxidation in mitochondria or stored in the form of neutral lipids to reduce their lipotoxicity [21]. Directing FAs to lipid droplets for storage in the form of TAGs has been shown to protect cells from lipotoxicity and prevent insulin resistance in various tissues, including the skeletal muscle, liver, and heart [27,47,140,141,142,143]. In fact, DGAT1-dependent lipid droplet formation mitigates lipotoxicity under various conditions of exogenous and endogenous lipid overload, including diet-induced obesity [144,145], cardiomyopathy [146,147], neuronal hypoxia [24], incubation with exogenous saturated and unsaturated FAs [9,27,144], and during periods of high autophagic flux [31]. Interestingly, even in adipose tissue, the lipolytic flux of FAs from lipid droplets into the cytosol causes cell damage and adipocytes undergo a “futile” ATP-driven cycle of FA release from TAGs by lipolysis and their re-esterification into TAGs, mediated by the DGAT1 enzyme, in order to reduce ER stress and adipose tissue inflammation [47].

Important evidence for the beneficial role of DGAT-mediated lipid droplet biogenesis at the organismal level has been obtained from studies focused on the connection between lipid accumulation and metabolic diseases. Overexpression of DGAT1 in white adipose tissue of mice exposed to high-fat diet leads to increased accumulation of TAGs in adipose tissue and to obesity, but it also improves insulin sensitivity in comparison with wild-type mice [145]. Similarly, mice overexpressing DGAT1 or DGAT2 in the liver develop steatosis but do not show any defects in insulin sensitivity and signalling, suggesting that lipid accumulation in the liver is not sufficient to cause insulin resistance [148]. Moreover, although intramyocellular fat deposits have been associated with insulin resistance, physical exercise elevates DGAT activity and TAG accumulation in skeletal muscle and reduces DAG and ceramide levels, a phenomenon referred to as “the athlete’s paradox” [149]. Furthermore, transgenic overexpression of myocellular DGAT1 reduces high-fat diet-induced insulin resistance in mice. Humans with severe heart failure have a marked reduction in FA oxidation and DGAT1 mRNA levels in the heart and accumulate DAG and ceramides [150]. In accordance, cardiomyocyte-specific DGAT1 knockout mice also accumulate toxic lipids, mostly DAG and ceramides, in the heart and have increased mortality due to heart failure [147]. It has also been shown that elevated DGAT1 expression in macrophages increases their capacity for TAG storage and protects from diet-induced insulin resistance and inflammation [144]. Moreover, the protective effects of antidiabetic PPARγ agonists against FA-induced inflammatory macrophage activation were dependent on DGAT1. In the endothelium, DGAT1-mediated lipid droplet biogenesis is important for buffering postprandial surges of FAs and reducing their lipotoxicity [151]. Therefore, in most tissues exposed to elevated levels of circulating lipids, lipid droplet biogenesis protects cells from lipotoxicity and mitigates the effects of lipid overload on the development of insulin resistance, inflammation, and metabolic disorders.

### 4.2. Lipid Droplets Mediate the Protective Effects of Unsaturated FAs Against Lipotoxicity 

In a seminal study, Listenberger et al. (2003) have shown that interfering with lipid droplet biogenesis by depletion of DGAT1 leads to cell death in fibroblasts exposed to the otherwise non-toxic oleate [27]. They found that palmitate is poorly incorporated into the TAG pool, whereas exogenously-added or stearoyl-CoA desaturase 1 (SCD1)-derived unsaturated FAs were readily incorporated into lipid droplets. Moreover, oleate reduced palmitate-induced apoptosis by channelling the saturated FA into lipid droplets and reducing ceramide and ROS production associated with its toxicity. Exogenous oleate also prevented palmitate-induced inflammation, insulin resistance, and apoptosis in skeletal muscle cells by channelling palmitate into lipid droplets and stimulating β-oxidation via AMP-activated protein kinase (AMPK) activation [152,153]. In human cardiomyocytes, acyl-CoA synthetase long-chain family member 1 (ACSL1) and PPARγ overexpression augmented TAG accumulation and alleviated ER stress induced by exogenous palmitate, suggesting that channelling palmitate to neutral lipid stores reduces its lipotoxicity [40]. On the contrary, knockdown of DGAT1 in cultured rat hepatocytes exposed to exogenous FAs did not enhance palmitate lipotoxicity or prevent the ability of oleate to reduce palmitate-induced cell damage [154]. In accordance, Robblee et al. found that oleate mitigates palmitate-induced ER stress in myeloid cells independently of DGAT1 activity or palmitate incorporation into TAGs. Instead, the flux of saturated FAs into phospholipids was responsible for activation of the ER stress sensor inositol-requiring kinase 1α (IRE1α) and the NLR family pyrin domain-containing 3 (NLRP3) inflammasome, and this was counteracted by oleate incorporation into phospholipids [155]. Thus, the beneficial effects of oleate may also be based on its competition against palmitate for esterification into phospholipids [154,155]. Therefore, although a number of studies have shown that lipid droplet biogenesis mediates the protective effects of unsaturated FAs against saturated FA-induced lipotoxicity, more research is warranted to confirm this concept and define the underlying mechanisms.

Studies describing the relationship between FA lipotoxicity and lipid droplet metabolism in cancer cells are scarce. The roles of different FA species in supporting lipid droplet accumulation and cell growth in different cancer cell types in different environmental conditions are also not clear. It is generally accepted that excess amounts of saturated FAs are toxic to cancer cells, while unsaturated FAs, such as oleate, are most often cytoprotective, induce proliferation, and even provide protection from long-term starvation in some cancer cells [7,17,156,157]. However, as discussed below, the protective role of oleate may not be universal to all cancer cells. It has been shown that palmitate and other saturated FAs induce apoptosis in different breast cancer cell lines and are not incorporated into TAGs as readily as unsaturated FAs [156,157]. On the contrary, oleate reduces starvation-induced apoptosis and mitigates the lipotoxicity of saturated FAs in highly invasive MDA-MB-231 triple-negative breast cancer cells, likely by channelling saturated FAs toward TAG synthesis [157]. However, although oleate induces lipid droplet formation in MDA-MB-231, MCF-7, and T-47D breast cancer cells, it suppresses serum starvation-induced cell death only in MDA-MB-231 cells and it is toxic to the less aggressive hormone-responsive T-47D cells [7,9]. Similarly, exogenous oleate induces lipid droplet biogenesis, but it does not provide a survival advantage to serum-deprived HeLa cells [158]. More studies are necessary to explain the basis for the different effects of oleate and other unsaturated FAs on lipid droplet accumulation and survival of different types of cancer cells, but it is likely that rather than the level of lipid droplet accumulation at a given time point, the balance between lipid droplet biogenesis/breakdown and other FA metabolic pathways that determine FA flux and availability will be critical determinants. The underlying mutational landscape and the specific metabolic alterations of different cancer cells most probably dictate the ability of unsaturated FAs and lipid droplets to protect or harm cells in particular microenvironmental conditions.

A number of studies have demonstrated that polyunsaturated fatty acids (PUFAs) have distinct and contrasting effects in cancer, with the pro-inflammatory ω-6 PUFAs, such as arachidonic acid (AA; 20:4, ω-6), mostly displaying pro-tumorigenic effects and ω-3 PUFAs, such as eicosapentaenoic (EPA; 20:5, ω-3) and docosahexaenoic acid (DHA; 22:6, ω-3), showing anti-tumorigenic, anti-inflammatory, and pro-apoptotic effects in cancer cells [159,160]. However, most in vitro studies have been performed with high micromolar, non-physiological concentrations of PUFAs [160,161,162,163] and often disregard the need for proper complexation of free FAs to serum albumin prior to application [164]. The total free (non-esterified) FA concentration in human plasma is approximately 200 μM after overnight fasting [165], with oleate being the most abundant FA (80 μM), followed by linoleate (15 μM), AA (3 μM), DHA (1 μM), and EPA (0.4 μM). Thus, cells exposed to high micromolar concentrations of PUFAs are under a massive overload of easily oxidizable FAs, suggesting that their apparent toxic effects may be largely exaggerated and may mask potential underlying effects. Indeed, we have recently found that both ω-3 and ω-6 PUFAs, used at low-micromolar, near physiological concentrations, display similar effects as oleate in inducing lipid droplet biogenesis and promoting breast cancer cell survival during stress [9]. As expected, higher micromolar concentrations of PUFAs, but not oleate or linoleate, induced oxidative stress-dependent cell death. Importantly, suppression of TAG synthesis by DGAT1 inhibition augmented PUFA-induced lipotoxicity, while stimulation of lipid droplet biogenesis reduced PUFA-induced cell death. The protective effect of lipid droplets was observed when their biogenesis was induced either by exogenous oleate, linoleate, or by the group X secreted phospholipase A_2_ enzyme (sPLA_2_). The enzyme binds very well to the phosphatidylcholine-rich plasma membrane of breast cancer cells and releases a mixture of mono- and polyunsaturated FAs, in which oleate and linoleate are by far the dominant FA species [9]. At first sight, these results suggest that the sPLA_2_, and its products oleate and linoleate, channel excess PUFAs into neutral lipid storage and thus prevent their oxidation and cell damage. Instead, we found that the enzyme enriches TAGs with FAs with low unsaturation levels and reduces the fraction of highly unsaturated PUFA-TAGs stored in lipid droplets. This effectively leads to “dilution” of the most oxidation-prone PUFAs within lipid droplets, reducing their availability for lipolytic release [9]. In accordance, the levels of PUFAs in the phospholipid pool were reduced. Moreover, suppression of TAG lipolysis by ATGL depletion reduced PUFA-induced oxidative stress and cell death, confirming that the release of PUFAs from lipid droplets is responsible for cell damage. Importantly, as described below (Section 7.3), a similar anti-lipotoxic and antioxidant role for lipid droplets has been recently described in vivo in *Drosophila* larvae exposed to hypoxia, whereby the sequestration of membrane-derived PUFAs in lipid droplets reduces their lipotoxicity and has a critical role in enabling neuronal cell proliferation during development [24]. Therefore, lipid droplet biogenesis, TAG acyl chain remodelling, and lipid droplet breakdown are all determinants of PUFA lipotoxicity, suggesting that differences in basal or stress-induced levels of these processes in cancer and other cell types may strongly influence the lipotoxic potential of PUFAs. The capacity of cancer cells to balance (poly)unsaturated FA sequestration and release from lipid droplets is thus important for their ability to cope with FA-induced lipotoxicity and to use FAs for cell survival. 

### 4.3. Lipid Droplets Also Store Acylceramides and Reduce Ceramide Accumulation-Induced Cell Damage

Interestingly, it was shown recently that acylceramides are also stored in lipid droplets, thus further expanding the roles of lipid droplets in their capacity to act as a sink for diverting not only lipotoxic FAs and DAGs, but also ceramides, from a bioactive to a storage pool [39]. It was found that acylceramides are synthesized by a complex involving ACSL5, ceramide synthase (CerS) and DGAT2 at the ER/lipid droplet interface in cultured cells and in the livers of mice on a high-fat diet. The conversion of ceramide into acylceramide and its sequestration into lipid droplets was associated with prevention of cell death. In colorectal carcinoma cells, stimulation of acylceramide biogenesis led to protection from ceramide-mediated 5-fluorouracil-induced cell death, whereas a blockade of acylceramide biogenesis led to elevated ceramide accumulation and apoptosis. Thus, the storage of acylceramide in lipid droplets in cancer cells may improve their resistance to chemotherapy by reducing pro-apoptotic ceramide levels. Interestingly, both DGAT1 and DGAT2 displayed ceramide acyltransferase activity, although DGAT2 is likely the predominant isoform responsible for acylceramide synthesis in vivo [39]. Thus, DGAT enzymes directly regulate the lipotoxicity of both DAG and ceramide by acylating and diverting these lipids into storage. Likewise, it may be anticipated that lipases that release ceramide from lipid droplets would also strongly impact the level of cell damage instigated by ceramide [39]. This previously unknown mechanism of reduction of ceramide toxicity calls for a re-evaluation of many previous studies on the lipotoxicity associated with saturated FA-induced ceramide and DAG accumulation. Thus, lipid droplets act as central anti-lipotoxic organelles that control FA, DAG, cholesterol and ceramide lipotoxicity by coordinating TAG, CE and acylceramide storage. 

### 4.4. Lipid Droplets Accumulate Cholesterol Esters to Regulate Cholesterol Availability and Promote Tumour Growth 

Although the majority of studies addressing the role of lipid droplets in cancer have focused on FA metabolism and TAG accumulation, recent reports suggest that CE accumulation in cancer cells is also associated with tumour growth. CE accumulation has been associated with a poor clinical outcome in breast cancer patients [126] and with the aggressiveness of glioblastoma, prostate, and pancreatic cancer [166,167,168]. Elevated accumulation of CEs in prostate cancer has been associated with upregulated PI3K/Akt signalling and an increased uptake of exogenous lipids [166]. Importantly, inhibition of cholesterol esterification impaired cancer cell aggressiveness and suppressed tumour growth in mouse xenograft models. In glioblastoma, inhibition of ACAT1 increased cholesterol levels, leading to inhibition of SREBP-1 and suppression of lipogenesis and tumour growth [168]. In a mouse model of pancreatic cancer, depletion of ACAT1 suppressed tumour growth and metastasis by increasing intracellular free cholesterol levels, causing elevated ER stress and cell death. A lipid accumulating phenotype has been described in highly invasive and tumourigenic triple-negative (ER–, PR–, HER2–) breast cancer cells and it has been associated with increased cell proliferation, migration and cell survival [7,17,169,170]. In comparison with less tumorigenic hormone-responsive breast cancer cells, triple-negative breast cancer cells accumulate significantly greater amounts of TAGs upon exposure to exogenous unsaturated FAs and display a superior ability to use lipid droplets and FA oxidation for cell survival during prolonged starvation [7,9,17]. These cells also display a greater uptake of both exogenous oleate and lipoproteins and have a higher expression of ACAT1 in comparison with less aggressive ER+ breast cancer cells. Importantly, the suppression of CE accumulation by inhibition of ACAT1 reduced low-density lipoprotein (LDL)-stimulated proliferation and migration [169,170]. Thus, although the underlying mechanisms have not been clarified, the ability of cancer cells to increase their storage of CEs in lipid droplets and thus remove cholesterol from its bioactive pool is beneficial for the progression of several aggressive types of cancer.

### 4.5. Lipid Droplets Protect Cancer Cells From Chemotherapeutic Drugs

Cancer drug resistance is a major obstacle in cancer therapy and lipid droplets may be important for the ability of cancer cells to survive chemotherapeutic stress [12,18]. Cotte et al. [16] have recently investigated the role of lipid droplets in colorectal cancer under chemotherapeutic conditions. They found that the common chemotherapeutic agents 5-fluorouracil and oxaliplatin induce lysophosphatidylcholine acyltransferase 2 (LPCAT2)-dependent lipid droplet accumulation. They found that LPCAT2-driven lipid droplet biogenesis protects cancer cells from chemotherapy-induced ER stress and cell death both in vitro and in vivo. The authors also show that lipid droplets sequester calreticulin and prevent its exposure on the plasma membrane, thus impairing CD8+ T cell infiltration and immunogenic cell death under chemotherapeutic treatment [16]. The sequestration of calreticulin to lipid droplets suggests that limiting the exposure of danger-associated molecular patterns (DAMPs) may be another important mechanism through which lipid droplets protect stressed cancer cells from cell death. Besides identifying LPCAT2 as a potential target to improve the efficacy of chemotherapy in colorectal cancer, this study also suggests that the presence of elevated levels of lipid droplets may be diagnostically useful to predict tumour chemoresistance.

## 5. Lipolysis and Lipotoxic Stress 

### 5.1. ATGL Regulates Fatty Acid-Mediated Energy Production, Signalling, and Lipotoxic Stress

Relatively little is known about the role of lipolytic lipid droplet breakdown in the regulation of lipotoxicity and cancer cell survival during stress [12,57]. Nevertheless, studies on the role of lipolysis and, in particular, ATGL, in cellular and organismal lipid homeostasis, provide important clues for cancer biology. ATGL catalyses the rate-limiting step in TAG lipolysis and is the major cytosolic TAG hydrolase in adipose tissue, the heart and liver, and other tissues [57,85,88,171]. The enzyme has a crucial role in supplying skeletal muscle with FAs from adipose tissue and is involved in the mobilisation of intramyocellular TAG reserves for energy conversion in the working muscle [86]. ATGL also enables FA transfer from lipid droplets to mitochondria in cardiomyocytes, hepatocytes, starved mouse embryonic fibroblasts, and, presumably, in most other cell types and tissues [25,66,88]. Lipolytic lipid droplet breakdown fuels the mitochondrial oxidative metabolism and enables cell survival during nutrient deprivation in various cells, including several cancer cell types [7,9,15,25]. Aside from being substrates for energy production, ATGL-derived FAs promote signalling pathways leading to an elevated mitochondrial and oxidative gene expression, thus, matching β-oxidation with FA mobilisation from lipid droplets [66,67,88,172]. ATGL deficiency in humans causes systemic TAG accumulation and neutral lipid storage disease with cardiomyopathy, but not obesity [173]. In accordance, mice with global ATGL deletion accumulate TAG in many tissues and exhibit lethal cardiac dysfunction due to a massive accumulation of TAG in the heart [174]. Paradoxically, ATGL-deficient mice are only moderately obese and display improved glucose tolerance and insulin sensitivity despite TAG accumulation in multiple tissues [174,175,176]. This unexpected adipose tissue phenotype was explained by a study using ATGL-deficient mice expressing ATGL only in the heart [177]. These mice were resistant to diet-induced obesity and had normal insulin sensitivity and adipogenic differentiation, however, they displayed impaired signalling through PPARγ and SREBP-1c. Therefore, reduced lipogenesis may, in fact, be responsible for the resistance of ATGL-deficient mice to high-fat diet-induced obesity and obesity-induced insulin resistance. In line with this, inhibition of ATGL in mice using Atglistatin leads to improvement of insulin signalling and a reduction of diet-induced obesity and hepatosteatosis, likely due to a reduced release of adipose tissue-derived FAs in the circulation [178]. Importantly, the pharmacological inhibition of ATGL did not cause ectopic lipid accumulation or cardiac dysfunction in mice even after long-term treatments. The inhibition of ATGL is thus a promising therapeutic strategy to treat obesity, metabolic disorders, and potentially also cancer [178]. 

While the prevailing role of ATGL in non-adipose tissues is most likely providing FAs for mitochondrial energy production and stimulating signalling associated with oxidative metabolism, TAG lipolysis may also lead to lipotoxicity. Excessive lipolysis is often associated with states of lipid overload and this may cause cell damage by increasing the pool of free FAs in the cytosol, altering signalling pathways that regulate oxidative metabolism, ER homeostasis, and stimulating β-oxidation and ROS production [53,54,55]. The inhibition of ATGL-mediated lipolysis by perilipin 5 provides a “lipolytic barrier” against uncontrolled TAG lipolysis in oxidative tissues, thus preventing cardiac, hepatic and muscle lipotoxic injury and insulin resistance [179,180,181,182,183]. Perilipin 5 couples TAG lipolysis with the metabolic demand for FAs, prevents ceramide accumulation and protects tissues against oxidative stress induced by excessive β-oxidation and FA peroxidation [89,139,180,184]. Additionally, perilipin 5 forms transcriptional complexes with PPARγ coactivator-1α (PGC-1α) and sirtuin 1 (SIRT1) and promotes the transcription of mitochondrial and oxidative genes in response to catecholamine-triggered signalling [185]. Similarly, the absence of ATGL protects mice treated with tunicamycin from hepatic ER stress and inflammation by inducing TAG accumulation and enrichment with oleate, thus shifting the balance in FA composition to reduce the flux of lipotoxic palmitate in the liver [90]. Moreover, stimulation of lipolysis by ATGL overexpression leads to ER stress in cardiomyocytes treated with oleate, which, in contrast to palmitate, does not cause ER stress in cells with basal levels of ATGL [40]. In accordance with these studies, ATGL depletion in breast cancer cells challenged with lipotoxic concentrations of PUFAs leads to lipid droplet accumulation and reduction of oxidative stress and cell death, suggesting that the sequestration of PUFAs in lipid droplets is beneficial for protection from PUFA lipotoxicity [9]. On the contrary, ATGL deficiency in macrophages leads to TAG accumulation and cell death associated with ER stress, elevation of ROS, and mitochondrial damage [186], suggesting that ATGL may also play a protective role against lipotoxicity. Thus, both excessive TAG accumulation and lipolysis may induce cell damage and the precise regulation of ATGL activity may be critical in some cases. Collectively, depending on the cell type, state and environmental conditions regulating cellular stress, ATGL-mediated lipolysis may be responsible for providing FAs for mitochondrial energy production, regulation of oxidative metabolism, and cell survival, but it may also cause lipotoxic cell damage. Notably, lipid droplet lipolysis and mitochondrial metabolism are coupled and regulated at multiple levels and their coordination is critical for the removal of toxic FA species and the prevention of lipotoxicity. 

### 5.2. ATGL and Cancer

The role of lipolysis in cancer has not been a subject of many studies to date [97]. Elevated expression of MAGL has been observed in several cancers and a pro-tumourigenic role has been suggested for the enzyme based on its ability to promote cancer aggressiveness by upregulating the endocannabinoid and FA signalling pathways [87,187,188]. On the other hand, there are conflicting reports about the role of ATGL in cancer [189]. ATGL depletion leads to growth inhibition in several cancer cell lines [190,191], suggesting a pro-tumourigenic role for the enzyme. Additionally, recent studies suggest that ATGL activity in both tumours and neighbouring adipose tissue contributes to the adipocyte-induced metabolic reprogramming and aggressiveness of breast, pancreatic, and ovarian cancer [102,103,104,192]. Activated lipolysis in omental fat adipocytes provides lipids for energy production in ovarian cancer cells and promotes their growth at the metastatic site [103]. ATGL is overexpressed in high-grade mammary tumours and breast cancer cell lines in vitro [104]. Breast cancer cells take up FAs released via lipolysis from neighbouring adipocytes, whereas ATGL activity in breast cancer cells enables FA translocation to mitochondria and stimulates the induction of uncoupled FA oxidation. The latter sustains AMPK activation and enables a persistent metabolic reprogramming that stimulates tumour invasion and metastasis [104]. However, recent in vivo studies suggest a tumour suppressor role for ATGL since its expression is reduced in several human cancers, including breast invasive adenocarcinoma, and it correlates with a reduced patient survival [193]. Importantly, mice lacking ATGL spontaneously develop lung tumours [193] and an adipose tissue-specific combined deficiency of ATGL and HSL leads to the development of liposarcoma [194]. Clearly, more studies are necessary to clarify the role of ATGL in cancer. However, in light of these conflicting reports, and given the pleiotropic roles of ATGL and lipolysis in different tissues in energy metabolism and beyond, a universal pro-tumourigenic or tumour suppressor role for ATGL should not be expected. Its role is most likely dependent on the particular type and stage of cancer, specific metabolic profile, nutrient status, and the level of environmental stress imposed by the presence or absence of nutrients and oxygen that critically affects lipid droplet metabolism. Importantly, the involvement of peritumoural adipocyte-derived ATGL in the provision of FAs for tumour growth suggests that ATGL may be a potential therapeutic target in tumours that depend on FAs derived from neighbouring adipose tissue. 

## 6. Lipid Droplets are Synthesized and Broken Down during Nutrient Stress to Enable Cell Survival

### 6.1. Lipid Droplet Biogenesis is Enhanced during Nutrient Deprivation 

Lipid droplet biogenesis is a general cellular response to exogenous lipid overload to reduce the accumulation of lipotoxic lipids, as described above. However, lipid droplet biogenesis also occurs in response to nutrient deprivation. This has been demonstrated in various types of cells exposed to complete or partial nutrient deprivation, such as primary neurons and astrocytes, CHO-K1 cells, HeLa cervical carcinoma, Huh7 liver carcinoma, U2OS osteosarcoma, LN18 glioblastoma cancer cells, and starved mouse embryonic fibroblasts (MEFs) [15,23,25,31,195]. Importantly, nutrient deprivation-induced lipid droplet biogenesis occurs even in the absence of exogenous lipids and is independent of endogenous de novo lipid synthesis. Rather, it is driven by the autophagic and/or phospholipase-mediated mobilisation of the available endogenous, structural lipids (Figure 3 and Figure 4) [23,25,31]. Paradoxically, cells mobilise endogenous lipids and engage in an ATP-consuming TAG synthesis process despite the absence of sufficient nutrients. Lipid droplet biogenesis must, therefore, be essential for cell survival, but it was not clear until recently why a futile cycle of FA esterification into TAG and lipolysis would be necessary for energy production and cell survival. Studies in starved MEFs have shown that structural lipids are mobilised through autophagy of membranous organelles and that lipid droplets are necessary for at least two main reasons: (1) to prevent the lipotoxicity of FAs released by autophagy into the cytosol [31] and (2) to enable efficient transfer of FAs from lipid droplets to the network of tubulated mitochondria for oxidation [25,31,34,41]. The lipid droplet biogenesis in starved MEFs depends on the activation of autophagy (see discussion in Section 8.3) and on DGAT1 activity. DGAT1 depletion or inhibition leads to mitochondrial damage due to FA overload and a concomitant accumulation of toxic acylcarnitine species. Thus, DGAT1-mediated lipid droplet biogenesis in starved cells is necessary to buffer autophagy-liberated FAs and prevent mitochondrial damage. 

Previous studies have suggested that endogenous FAs required for TAG synthesis may also be derived from phospholipase A_2_ (PLA_2_)-mediated phospholipid hydrolysis (Figure 4). Namely, TAG synthesis in nutrient-deprived CHO-K1 cells is dependent on the activity of calcium-independent group VIA PLA_2_ (also referred to as iPLA_2_β and PNPLA9; encoded by the *PLA2G6* gene), an enzyme traditionally ascribed with a role in membrane remodelling and repair [196,197]. Interestingly, the process of lipid droplet biogenesis, but not TAG synthesis, required the subsequent phosphorylation and activation of another PLA_2_ enzyme—the calcium-dependent cytosolic group IVA PLA_2_ (also cPLA_2_α; encoded by the *PLA2G4A* gene)—which is most likely responsible for facilitating lipid droplet nucleation by regulating ER membrane shape and curvature [23,76,112,198]. Interestingly, besides being implicated in the regulation of the formation of the organelle [76,112], several other PLA_2_s [7,9,112,199] and phospholipases D (PLD) [24] have been implicated in channelling phospholipid-derived FAs into TAGs and CEs for lipid droplet biogenesis (Figure 4). Since a comprehensive study addressing the involvement of autophagy and PLA_2_s in the provision of FAs for lipid droplet biogenesis in starved cells has not been performed, it is possible that PLA_2_s act, in fact, as part of the broader autophagy-induced process of lipid droplet biogenesis. Phospholipase activity-derived FAs could be preferred over autophagy when a particular phospholipid and TAG acyl-chain remodelling is required (see Section 7.3), instead of the bulk FA provision by the lysosomal degradation of membranous organelles. In such cases, phospholipase-derived FAs could provide a subtler and safer channelling of FAs from membrane phospholipids to TAGs, simultaneously optimizing membrane saturation to adapt to cellular stress and providing FAs for TAG synthesis and energy production in mitochondria. It is thus possible that, depending on the conditions and cell type in question, autophagy and/or phospholipase activity may participate in lipid droplet biogenesis during stress. 

### 6.2. Lipid Droplet Breakdown is Necessary for Cell Survival during Starvation

Several lines of evidence suggest that FAs stored in starvation-induced lipid droplets are mobilised by cytoplasmic lipases, and not lipophagy (see also Section 8.3), to fuel the mitochondrial oxidative metabolism and to enable cell survival during nutrient deprivation [9,15,25,31]. The transfer of FAs from lipid droplets to mitochondria in acutely starved MEFs is dependent on ATGL and is inhibited by etomoxir, an inhibitor of carnitine palmitoyltransferase 1A (CPT-1A) and of FA translocation into mitochondria [25]. Furthermore, a close spatial proximity of lipid droplets and tubulated mitochondria is required for efficient transfer and redistribution of FAs within mitochondria [25,41]. This requirement for precise channelling of FAs from lipid droplets to mitochondria may explain why lipolysis, rather than lipophagy, is the preferred process for FA transfer to mitochondria in Hanks’ balanced salt solution (HBSS)-starved cells [25]. However, under milder and prolonged starvation conditions, lipophagy may be activated and may become the predominant mechanism of lipid droplet breakdown [25,51,62]. Interestingly, in serum-starved MEFs, autophagy-driven lipid droplet biogenesis does not occur [31], but lipophagy contributes to the lipid droplet breakdown [25]. We have found that while ATGL is involved in TAG lipolysis in breast cancer cells exposed to prolonged starvation, its depletion is not sufficient to fully prevent lipid droplet breakdown and significantly reduce the pro-survival effect of lipid droplets [9], suggesting the involvement of other lipases and/or lipophagy in serum starvation-induced lipid droplet breakdown in breast cancer cells. Therefore, the importance of autophagy in lipid droplet biogenesis and the predominant role of lipophagy vs. lipolysis in lipid droplet breakdown most likely depends on the cell type, its particular metabolic requirements, and the type and length of nutrient stress imposed. 

## 7. Lipid Droplets and Oxidative Stress

Cancer cells display elevated rates of reactive oxygen species (ROS) production due to oncogenic mutations, loss of tumour suppressors, accelerated metabolism necessary to sustain rapid growth, and adaptation to the various stresses imposed by the tumour microenvironment (i.e., nutrient and oxygen deprivation, infiltrating immune cells) [200,201]. Cancer cells may thus experience oxidative stress during both oncogene-driven nutrient excess and rapid metabolism and during nutrient and/or oxygen deprivation in poorly vascularized tumours [1]. Importantly, it is now clear that ROS- induced pro-tumourigenic signalling pathways are necessary for cancer cell survival, growth, proliferation, and metastasis. High levels of ROS, in turn, oxidize other molecules, such as proteins, lipids, and DNA, thus, further supporting oncogenic transformations. However, prolonged exposure to elevated ROS may lead to oxidative stress-dependent cell death and cancer cells simultaneously upregulate antioxidant pathways in order to maintain redox homeostasis. Oxidative stress also affects cellular lipid metabolism on several levels. The availability of glucose carbons for lipogenesis is highly restricted during hypoxia and cancer cells shift to a reductive glutamine metabolism or cytosolic acetate carboxylation for lipid synthesis [94,202,203]. Hypoxic cancer cells may upregulate FA uptake to compensate for impaired de novo lipogenesis [5,8]. Moreover, hypoxia inhibits FA oxidation and can induce the formation of lipid droplets as a temporary storage of energy for use during reoxygenation [8,204]. However, during metabolic stress, FA oxidation maintains the production of NADPH and is critical for protection against oxidative stress and cancer cell survival [95,101]. On the contrary, upregulated lipolysis and FA oxidation may also result in an elevated production of ROS and cell damage [53,180,205]. Imbalances in redox homeostasis are thus associated with significant alterations in lipid metabolism and it is not surprising that they also affect the lipid droplet metabolism. In this section, we discuss how lipid droplets support the cellular response to oxidative stress, and conversely, how hypoxia/oxidative stress regulates lipid droplet biogenesis and degradation. 

### 7.1. Lipid Droplets Accumulate in Hypoxia and Protect Cancer Cells from Oxidative Stress

Tumour cells often face restricted access to nutrients and oxygen due to an aberrant vascularization and poor blood supply [206]. Hypoxia, a non-physiological level of low oxygen tension, is a common feature of most solid and large tumours [207]. The hypoxic tumour microenvironment affects cancer metabolism by upregulating hypoxia-inducible factor (HIF) signalling, thereby promoting angiogenesis, metastasis, aggressiveness, chemoresistance, resistance to radiotherapy, and a poor overall outcome [208,209]. Short- and long-term exposure to hypoxia has been shown to promote cell survival by activating cellular stress response mechanisms—such as autophagy—and enhance tumour aggressiveness [210,211]. Hypoxia-associated induction of lipid droplet biogenesis has been observed in different cancers, including brain, breast, renal, and prostate cancers [8,12,26,28,29,204,212]. Studies in C6 rat models of glioma have shown that lipid droplets accumulate in the perinecrotic and necrotic hypoxic areas of the tumour core [212,213]. Primary glioma cultures obtained from patients also accumulate lipid droplets and have an increased expression of PPARα [204]. Hepatocellular carcinoma and cervical adenocarcinoma cells exposed to hypoxia accumulate lipid droplets through a direct HIF-1α-dependent stimulation of lipin 1 expression, a phosphatidate phosphatase involved in the production of DAG [28]. Constitutive HIF signalling and abundant lipid droplet accumulation are hallmarks of clear-cell renal carcinoma and it was shown recently that HIF2α-dependent perilipin 2 (PLIN2) expression stimulates lipid droplet accumulation, protects from ER stress, and promotes cell proliferation and xenograft tumour growth [26]. Several studies have shown that hypoxia leads to a blockade in the endogenous production of unsaturated FAs due to the inhibition of oxygen-dependent SCD1-mediated FA desaturation and that it renders cancer cells dependent on the uptake of unsaturated FAs to reduce hypoxia-induced ER stress and prevent cell death [4,5,107]. In accordance, glioblastoma and breast cancer cells exposed to hypoxia shift from de novo lipogenesis to FA uptake and accumulate lipid droplets, which are necessary to prevent ROS accumulation and to enable cancer cell survival [8]. Lipid droplet biogenesis was dependent on the HIF-1α-mediated upregulation of fatty acid-binding proteins (FABPs) 3 and 7, members of a family of proteins involved in FA uptake, metabolism and transport. Knockdown of FABP3, FABP7 or of the structural lipid droplet protein PLIN2 significantly impaired lipid droplet biogenesis during hypoxia, reduced NADPH levels, and increased ROS accumulation, suggesting that FA uptake and lipid droplet biogenesis are crucial for redox homeostasis. Accordingly, exogenous addition of the unsaturated FA oleate also protected cells from ROS toxicity. Importantly, the inhibition of lipid droplet formation by depletion of FABP3/7 led to suppression of xenograft tumour growth in vivo. Thus, lipid droplet biogenesis in hypoxia is most likely a protective mechanism that promotes cancer cell survival under oxygen-deprived conditions by reducing ROS accumulation. The accumulated lipid droplets provide a temporary storage of FAs during hypoxia and release them after reoxygenation to enable ATP production and cell growth [8]. Similarly, hypoxia-induced changes in lipid mobilisation have also been associated with increased TAG accumulation in prostate cancer cells and in extracellular vesicles released by these cells. Following reoxygenation, lipid droplets provided FAs for β-oxidation and enabled cancer cell growth [29]. Collectively, the studies described above show that cancer cells exposed to hypoxia upregulate FA uptake and accumulate lipid droplets, which enable ER and redox homeostasis during oxygen deprivation and provide FAs for mitochondrial energy production to boost cell growth following reoxygenation. 

### 7.2. Excessive Lipolysis is Damaging to Hypoxic Cancer Cells 

Recent studies have established that hypoxia affects the expression of several lipid droplet-associated proteins involved in lipid droplet breakdown [205,214]. Gimm et al. have shown that hypoxia-activated HIF-1-mediated signalling upregulates the expression of hypoxia-inducible lipid droplet-associated protein (HILPDA)/hypoxia-inducible gene 2 (HIG2)—a protein localized on the lipid droplet surface—in a number of cell lines and mouse tissues in vivo [214]. HIG2 binds to the surface of lipid droplets and its overexpression promotes neutral lipid accumulation in HeLa cells. Furthermore, HIG2 co-localization with lipid droplets was observed in HIF-1α-positive areas in renal cell carcinoma samples, suggesting that HIG2 may be used as a marker of hypoxia-induced lipid droplets [214]. Moreover, HIG2 expression is elevated during anti-angiogenic treatment and it may promote tumour resistance to therapy [215]. Recently, the basis for the HILPDA/HIG2-induced lipid droplet accumulation was explained by studies from two laboratories showing that the protein is a direct inhibitor of ATGL [205,216]. Zhang et al. found that the observed downregulation of ATGL-mediated lipolysis during hypoxia is due to HIF-1-induced upregulation of HIG2 [205]. Importantly, HIG2 depletion enhanced lipid droplet breakdown and β-oxidation, leading to elevated ROS production and impaired xenograft tumour growth. Depletion of ATGL reversed the effects of HIG2 knockout, suggesting that HIG2-mediated ATGL inhibition enables the survival of hypoxic cancer cells by reducing lipid droplet degradation and sequestering FAs away from mitochondrial oxidative and ROS-producing pathways [205]. Thus, ATGL-mediated lipid droplet breakdown is damaging to cancer cells during hypoxia and the ability of cancer cells to modulate lipolysis may determine their potential to survive oxygen deprivation. Other inhibitors and activators of ATGL, such as perilipin 5, G0/S2 switch regulatory protein 2 (G0S2), and abhydrolase domain-containing protein 5 (ABHD5/CGI-58), whose roles in cancer are currently under investigation [189,190,191,217,218], could have an important impact on lipid droplet metabolism in hypoxic cancer cells. As discussed above, based on its involvement in the protection of several tissues from lipolysis-induced oxidative stress, including ischemia/reperfusion injury in the heart [180], it will be particularly interesting to find out whether perilipin 5 is involved in the survival of some cancer cell types exposed to fluctuating levels of oxygen in the tumour microenvironment.

### 7.3. Sequestration of PUFAs in Lipid Droplets Reduces Membrane Lipid Peroxidation and Protects Cells from Oxidative Stress 

Besides hypoxia and the accumulation of endogenous lipid metabolites that disrupt mitochondrial and ER homeostasis, such as saturated FAs, ceramides, and DAG, oxidative stress can also arise due to chemotherapeutic drugs, exogenous pro-oxidants, and the accumulation of oxidized PUFAs. PUFAs are significantly more sensitive to oxidation in comparison to saturated and monounsaturated FAs. Cell membrane phospholipids containing PUFAs are particularly vulnerable to lipid peroxidation chain reactions during oxidative stress, thereby further amplifying ROS production [219]. Accordingly, increasing the relative degree of membrane (poly)unsaturation by inhibition of de novo lipogenesis sensitizes cancer cells to lipid peroxidation and oxidative stress-induced cell death [106]. Recently, an antioxidant role for lipid droplets against PUFA-induced lipotoxicity and oxidative stress was reported in neuronal and cancer cells [9,24]. In *Drosophila* larvae exposed to oxidative stress, it was shown that lipid droplet biogenesis occurs in niche glial cells to prevent peroxidation of membrane PUFAs by redirecting them from membrane phospholipids to TAGs stored within lipid droplets [24]. This mechanism spares both glia and neuroblasts from hypoxia-induced oxidative stress and allows neuroblast proliferation. Interestingly, lipid droplet biogenesis was not dependent on HIF-1 and could be induced by exposing larvae to various pro-oxidants, suggesting that ROS are the main inducers of lipid droplets during both pro-oxidant treatment and hypoxia. In agreement, studies from another laboratory have shown that oxidative stress in neurons leads to ROS-dependent accumulation of lipid droplets in neighbouring glial cells [220,221]. Notably, both hypoxia and pro-oxidants induced PUFA redistribution from plasma membranes to TAGs where the PUFAs were less vulnerable to peroxidation than in membranes [24]. The redistribution was dependent on PLD, lipin 1, and DGAT1 enzymes, suggesting that FAs are transferred from membrane phospholipids via PLD-dependent production of phosphatidic acid, its dephosphorylation to DAG by lipin 1 and a final DAG acylation mediated by DGAT1 (Figure 4) [24]. Lipid droplets also protected cellular proteins from harmful products of peroxidation, such as 4-hydroxynonenal (4-HNE), suggesting an even wider antioxidant role for the organelle. Thus, hypoxia and oxidative stress induce lipid droplet accumulation in glial cells to sequester oxidation prone PUFAs away from oxidative pathways, thereby reducing stress and enabling the proliferation of neighbouring neuronal stem cells. As discussed in more detail above (Section 4.2), a similar antioxidant role of lipid droplets in aggressive breast cancer cells exposed to lipotoxic levels of PUFAs has been recently described [9]. These cells benefited from sequestration of PUFAs in lipid droplets and both sPLA_2_-mediated stimulation of lipid droplet biogenesis, as well as suppression of ATGL-mediated lipolysis, protected cells from PUFA-induced oxidative stress. sPLA_2_-induced protection from PUFA lipotoxicity was associated with extensive TAG remodelling and reduced levels of PUFAs present in cellular membranes, revealing an important connection between TAG and phospholipid remodelling for cancer cell resistance to stress [9]. Interestingly, an enrichment of PUFA-TAGs in lipid droplets has been observed during the activation of hepatic stellate cells [222,223], a process critical for the development of liver fibrosis and associated with elevated levels of oxidative stress [224]. Moreover, fasted mice have elevated levels of very long-chain PUFA-TAGs in their hepatocyte lipid droplets in comparison with mice fed a high-fat diet [225]. Cisplatin treatment has been associated with an elevated accumulation of oleate and linoleate in lipid droplets of cancer cells [226]. An enrichment of TAGs with PUFAs has also been found in visceral adipose tissue samples from colon cancer patients, along with elevated expression of markers of inflammatory lipid metabolism, including phospholipases and prostaglandin synthesis–related enzymes, such as the group X sPLA_2_ and cyclooxygenase-2 (COX-2) [227]. Lipid droplet biogenesis is also induced during apoptosis [36,228] and PUFA-TAG enrichment has been suggested as a general feature of apoptosis [37]. Thus, the sequestration of PUFAs in lipid droplets and away from membrane phospholipids may be a general cytoprotective mechanism activated during various conditions associated with oxidative stress, to prevent further damage due to membrane lipid peroxidation. It will be interesting to see in future studies whether similar PUFA sequestration mechanisms protect cancer cells from various stress conditions characterized by an elevated ROS production. 

## 8. Lipid Droplets and Autophagy/Lipophagy

Autophagy is an evolutionarily conserved catabolic process of recycling dispensable cytoplasmic material by lysosomal degradation, thus providing building blocks and metabolic substrates for processes essential for cell survival [229,230,231]. Under physiological conditions, constitutive autophagy maintains cellular homeostasis by degrading potentially toxic products of normal cellular metabolism, such as damaged mitochondria and protein aggregates. Importantly, autophagy is crucial for the ability of both normal and malignant cells to cope with stress and adapt to adverse environmental conditions, such as nutrient and oxygen deprivation [229,232]. Autophagy is activated under various stressful conditions, including nutrient deprivation, hypoxia, elevated ROS levels, and, interestingly, by lipid overload and exogenous unsaturated FAs [129,233,234]. It acts as a tumour suppressor by preventing the accumulation of damaged cellular components, but cancer cells also use autophagy to survive stressful conditions, such as nutrient deprivation, hypoxia, and the high metabolic demand during rapid proliferation. Autophagy has thus been under investigation as an attractive therapeutic target in efforts to reduce tumour growth and chemoresistance. However, autophagy also promotes tumour-specific immune responses and recent studies suggest that the activation of autophagy using safe nutritional interventions may, in fact, improve the efficacy of anticancer agents [233].

There are three main types of autophagy and they are all involved in lipid droplet metabolism: macroautophagy, microautophagy, and chaperone-mediated autophagy (CMA). Macroautophagy (hereafter named “autophagy”) refers to the packaging of cytosolic cargo into an autophagosome and its fusion with the lysosome for enzymatic breakdown of the engulfed material. Although initially considered a non-selective process, several types of selective autophagy have been discovered [230] and named according to their substrate specificity: protein aggregates (aggrephagy), peroxisomes (pexophagy), mitochondria (mitophagy), ER (reticulophagy), pathogens (xenophagy), lysosomes (lysophagy), and lipid droplets (lipophagy) [235]. CMA is a specialized form of selective autophagy, so far characterized only in mammalian cells, and includes a motif-based recognition of damaged proteins by the heat shock cognate protein of 70 kDa (Hsc70) and lysosomal intake mediated by the lysosome-associated membrane protein type 2 (LAMP-2A) [236]. In contrast, microautophagy enables the uptake of cytoplasmic material by a direct invagination of the lysosomal membrane and it has been characterized in yeast [46,237], while its importance in mammalian cells is still questionable. 

**Figure 5:**
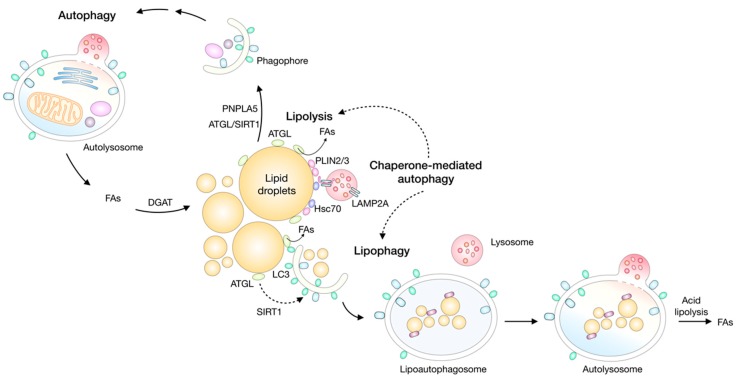
Crosstalk between autophagy and lipid droplets. Autophagy provides fatty acids (FAs) for lipid droplet biogenesis in starved cells, but it may also participate in lipid droplet breakdown though lipophagy. Lipid droplets promote autophagy through several possible mechanisms: acting as sites for autophagy initiation, providing lipids for the assembly of the autophagic machinery (e.g., via patatin-like phospholipase domain-containing 5 (PNPLA5)-mediated TAG lipolysis), maintaining endoplasmic reticulum (ER) homeostasis that enables autophagy initiation or stimulating sirtuin 1 (SIRT1) signalling by adipose triglyceride lipase (ATGL) that promotes both autophagy and lipophagy. Chaperone-mediated autophagy (CMA) promotes both lipophagy and ATGL-mediated lipolysis by degrading the lipid droplet-coating proteins perilipin 2 and 3 (PLIN2 and PLIN3), thereby freeing the access to the lipid droplet surface for lipases such as ATGL and for the lipophagic machinery. CMA is executed following recognition of PLINs by the chaperone Hsc70, their unfolding, and translocation into the lysosome through the lysosomal membrane glycoprotein receptor LAMP-2A. A coordinated regulation of lipophagy and lipolysis has also been suggested through a direct interaction between microtubule-associated protein light chain 3 (LC3) and ATGL. DGAT, diacylglycerol acyltransferase.

Recent studies have established strong links between autophagy and the maintenance of cellular and whole-body lipid homeostasis under physiological conditions and during stress, including that induced by lipid imbalance. The relationship is bidirectional since lipids also regulate autophagy. Lipid overload affects autophagic activity in multiple tissues, which has important implications for diseases associated with excessive lipid accumulation, such as metabolic disorders and cancer [51,129]. Interestingly, while short-term high-fat feeding induces autophagy in pancreatic β-cells to protect from lipotoxicity [129,238], long-term, chronic lipid overload impairs autophagic flux in β-cells, hepatocytes, and cardiomyocytes [129]. The crosstalk between autophagy and lipid metabolism is also reflected in their common hormonal, nutrient, and transcriptional regulators, such as mTORc1, AMPK, PPARα, PGC-1α, TFEB, TFE3, FoxO1, CREB, SREBP, and FXR [52,83,239,240]. Since lipid droplets are central to lipid homeostasis, there are also multiple ways of crosstalk between lipid droplets and autophagy (Figure 5). Autophagy may participate in lipid droplet biogenesis [25,31,34,241,242] and breakdown [62], but lipid droplets may also promote autophagy through several mechanisms [58,59,60,61]. Indeed, recent studies have shown that lipid droplets may be sites for autophagy initiation or provide lipids for the assembly of the autophagic machinery. Thus, autophagy and lipid droplet metabolism are connected through a number of different mechanisms, and the prevalence of each may depend on several factors, including the cell type in question, its nutritional status, and environmental context. Future studies will help us decipher the complex associations between these two important processes, thereby providing new clues for the understanding of metabolic diseases and cancer. In the following text, we have attempted to give a broad overview of the mechanisms of crosstalk between autophagy and lipid droplet metabolism. The detailed molecular mechanisms and pathophysiological aspects of autophagy/lipophagy have been reviewed recently in excellent reviews and will not be covered here [229,232,243,244].

### 8.1. Lipophagy is an Important Pathway for Lipid Droplet Breakdown during Stress

The role of autophagy in lipid droplet catabolism has been widely studied in the past decade after the surprising discovery of lipophagy. Indeed, besides lipolysis, lipophagy is emerging as a major pathway of lipid droplet breakdown in multiple tissues and pathophysiological settings [51]. Lipolysis is catalysed by neutral lipases acting on the surface of lipid droplets, whereas lipophagy uses the autophagic machinery to deliver lipid droplets or parts of lipid droplets to the lysosome for degradation by acidic lipases. Lipolysis predominantly affects TAGs, DAGs, and CEs, leading to shrinkage of lipid droplets, which is accompanied by remodelling of surface phospholipids and proteins. On the contrary, lysosomal lipid droplet breakdown degrades all storage and structural lipid droplet lipids and associated lipid droplet proteins, thereby releasing FAs, cholesterol, amino acids, and other products into the cytosol. Recent evidence suggests that the prevalent mechanism of lipid droplet breakdown depends on physiological requirements, nutrient status, and the type of stress but also that a significant cross-talk exists between lipolysis and lipophagy [51,56,91]. A number of studies have reported pivotal roles of lipophagy in the maintenance of lipid stores, the regulation of nutrient and energy balance, protection against oxidative stress, ER stress and lipotoxicity, protein quality control and even viral replication [48,49,50,51,52,245]. These and other studies have implicated lipophagy in important pathophysiological processes such as embryogenesis, lipid digestion in the gut, neural control of food intake and organismal energy balance, obesity, metabolic disorders, liver disease, atherosclerosis and cancer. 

The discovery of lipophagy in 2009 was based on findings that autophagy regulates lipid metabolism in mouse hepatocytes in vitro and in vivo [62]. Singh et al. showed for the first time that lipid droplets interact with the autophagic machinery during nutrient deprivation and that the inhibition of autophagy leads to TAG and lipid droplet accumulation. Depletion of the essential autophagic protein autophagy related 5 (Atg5) reduced TAG breakdown and β-oxidation rates in starved and oleate-fed cells indicating that autophagy regulates mitochondrial oxidation by providing free FAs from TAG hydrolysis. In line with this, autophagic deficiency leads to impairment of mitochondrial energy production and to oxidative stress-dependent cell death in hepatocytes [246]. Importantly, the study by Singh et al. [62] suggests that rather than de novo lipid synthesis, the dysfunction of lipophagy may contribute to elevated lipid accumulation in diseases associated with lipid storage in the liver. Namely, they found that a high-fat diet-induced lipid accumulation in the liver interferes with the execution of lipophagy, revealing a detrimental cycle in which elevated lipid storage reduces lipid clearance by autophagy, leading to a further increase in lipid accumulation. In accordance, it has been suggested recently that lipophagy facilitates the clearance of toxic ceramides arising from elevated de novo sphingolipid synthesis in the livers of obese mice [247]. Moreover, hormonal stimulation of autophagic activity reduces lipid droplet accumulation and prevents hepatotoxicity in mice on a high-fat diet [248], whereas both lipophagy and mitophagy protect against ethanol-induced liver injury [249]. These findings suggest that constitutive lipophagy may protect the liver against lipotoxicity by reducing the accumulation of toxic lipids, sustaining mitochondrial energy production and maintaining redox homeostasis. 

In contrast to its importance in liver metabolism, the role of lipophagy in other tissues is less defined. Current findings have not yet explained the role of lipophagy in adipocytes, mostly because of difficulties in differentiating between the effects of autophagy and lipophagy, but a protective role of lipophagy against obesity-induced inflammation and lipotoxicity has been proposed [129]. In accordance, stimulation of lipophagy by a caloric restriction mimetic in differentiated adipocytes leads to lipid droplet breakdown and a reduction of TAG levels associated with the activation of AMPK [250]. In response to atherogenic lipoprotein accumulation, lipophagy reduces the accumulation of CE-rich lipid droplets in macrophage foam cells [251] and it has been recognized as a potential target in preventing atherosclerosis and cardiovascular diseases [252]. Enhanced lipophagy has been shown to regulate lipid droplet turnover in enterocytes exposed to alimentary lipid micelles, likely to protect cells against lipotoxicity and ER stress [45,253]. Lipophagy also maintains energy homeostasis in the kidney during prolonged starvation by providing FAs for energy production in mitochondria [254]. Intriguingly, the packaging of circulating FAs into lipid droplets and lipophagy are necessary for channelling exogenous FAs into mitochondria and energy production in starved kidney proximal tubular cells. In hypothalamic neurons, lipophagy has been implicated in the regulation of food intake and energy balance by regulating the release of endogenous FAs that modulate neuropeptide levels and affect signalling pathways controlling food intake [255]. Moreover, autophagy in the central nervous system activates lipophagy and lipolysis in peripheral tissues as a response to cold exposure [256]. The induction of forced lipophagy in mouse embryos reduces lipid droplet levels during development and results in retardation, suggesting a novel role of lipid droplets and lipophagy during embryogenesis [257]. A sudden depletion of glucose in yeast cells activates AMPK-dependent lipid droplet breakdown by microautophagy, i.e., microlipophagy, and provides energy for long-term survival [258]. Microlipophagy has also been implicated in the management of ER lipid and protein quality control during phospholipid imbalance-induced ER stress [46,237]. It was found that lipid droplets are formed at ER aggregates and act as carriers for the transfer of damaged proteins and toxic lipids from the ER to the vacuole through microlipophagy [46,237]. Whether microautophagy occurs in mammalian cells is still unknown, but an alternative pathway of endosomal autophagy shares similar characteristics [259] and has a physiological role in erythropoiesis in vivo [260]. In summary, lipophagy is involved in important pathophysiological processes in various tissues by regulating lipid mobilisation, energy production, and lipotoxic cell damage, but its exact roles and the underlying mechanisms are yet to be defined.

The role of lipophagy in cancer has not been addressed by many studies so far. While several reports have indicated tumour promoting effects of lipophagy [261,262], most studies so far suggest that lipophagy restricts tumourigenesis [263,264,265,266]. Microtubule-associated protein 1S (MAP1S)-mediated lipophagy promotes lipid droplet clearance and higher levels of MAP1S have been associated with reduced tumour growth and metastasis and an increased patient survival in clear-cell renal cell carcinoma [263]. Another study has shown that overexpression of ATG14 facilitates lipid droplet breakdown in Hela cells and induces free FA accumulation leading to ER stress- and ROS-mediated apoptosis, whereas suppression of lipophagy by 3-methyladenine (3-MA) or inhibition of lysosomal acid lipase (LAL) reverses these effects [264]. LAL catalyses both TAG and CE hydrolysis in the lysosome and its deficiency or inhibition leads to several pathologies associated with elevated lysosomal lipid accumulation in mice and humans [56]. Recent evidence suggests that LAL plays a tumour suppressor role and its deficiency in mice has been linked with spontaneous tumourigenesis. Importantly, LAL re-expression in hepatocytes prevents liver metastases [265] and its reintroduction in the lung reduces inflammation and metastasis in lung cancer [266]. Interestingly, it was found recently that adipocyte-derived FAs upregulate FA oxidation and stimulate AMPK-dependent autophagy in neighbouring cancer cells [267]. Inhibition of autophagy suppressed lipid droplet breakdown and attenuated the growth-promoting effects of adipocytes, suggesting that adipocytes stimulate lipophagy in cancer cells to foster energy production and enable cancer cell survival. Finally, recent studies suggest that the tumour suppressor protein p53 stimulates lipid catabolism by regulating the expression of several autophagy and lipid metabolism genes, thereby enhancing lipophagy and FA oxidation [268,269,270]. Thus, current findings on the involvement of lipophagy in cancer provide mixed conclusions and more studies are necessary to delineate its roles. In analogy with the contrasting roles of lipolysis and ATGL in cancer and cell stress, as discussed above, it is likely that the role of lipophagy will be determined by the metabolic and oncogenic dysregulation of particular cancer cells and their exposure to stress.

### 8.2. Crosstalk between Lipolysis and Lipophagy/Autophagy

CMA has been recently described as a regulator of lipid droplet breakdown by acting as an activator of both lipolysis and lipophagy (Figure 5) [271]. In nutrient-deprived conditions, CMA facilitates the removal and lysosomal degradation of the lipid droplet-associated PLIN2 and PLIN3 proteins, thus, enabling lipid droplet surface access to ATGL and the autophagic machinery to initiate lipolysis and/or lipophagy. In principle, CMA may thus regulate both macro- and microautophagy-mediated lipid droplet breakdown as well as lipolysis mediated by neutral lipases [83]. Targeted deletion of the LAMP-2A CMA receptor in murine liver revealed that defective CMA causes hepatic glycogen depletion and steatosis and compromises the adaptation to energetic requirements under nutrient stress [272]. Additionally, AMPK-mediated PLIN2 phosphorylation was required for CMA-mediated PLIN2 degradation, revealing a new role of AMPK in stimulating lipid droplet breakdown during starvation [273]. The role of CMA in the adaptation of cancer cells to stress is currently unknown, but elevated CMA activity has been recently associated with increased growth and metastasis of breast cancer cells in vitro and in vivo [274]. Future studies will provide insights into the potential connections between CMA and lipid droplet metabolism in regulating the cancer cell stress response. 

A coordinated regulation of lipophagy and lipolysis has also been suggested through a direct interaction between microtubule-associated protein light chain 3 (LC3) and ATGL (Figure 5). Cold exposure induces autophagy in the central nervous system, which, in turn, activates both lipolysis and lipophagy in peripheral tissues, such as the brown adipose tissue and liver [256]. Interestingly, an interaction between LC3 and ATGL was observed after cold exposure in peripheral tissues and it was necessary for efficient lipolysis, suggesting that LC3 regulates lipolysis. Thus, autophagosomal membranes and proteins may serve as scaffolds for regulating the recruitment and activity of neutral lipases, ensuring a coordinated regulation of lipolysis and lipophagy [256].

The crosstalk between lipid droplets and autophagy/lipophagy also extends to recent discoveries, suggesting that lipid droplets are necessary for the proper initiation and execution of autophagy (Figure 5). The consumption of lipid droplets during starvation has been associated with lipid droplets not as substrates for lipophagy, but rather as sources of lipids for the biogenesis of nascent autophagosomal membranes [58]. The lipid droplet-localized TAG lipase patatin-like phospholipase domain-containing 5 (PNPLA5) was necessary for autophagy, suggesting that TAG lipolysis contributes to autophagosomal formation [58]. Studies in yeast have shown that lipid droplet lipids are required for starvation-induced formation of autophagosomes and that several TAG and sterol ester lipases are essential for autophagy [59,275]. However, another study has shown that lipid droplets may be dispensable as a source of lipids for autophagosome formation but are instead necessary for buffering FA fluxes and the maintenance of ER homeostasis that is, in turn, critical for autophagosome biogenesis [60]. The mechanisms that determine the source of lipids for autophagic initiation are still unknown, but these studies suggest that lipid droplets may be required for autophagy in multiple ways, acting as sources of essential lipids for autophagosomal membrane biogenesis and/or as regulators of lipid trafficking that is critical for ER homeostasis and thus indirectly affecting autophagy.

Furthermore, it has been shown recently that lipolysis mediated by ATGL (PNPLA2) may stimulate autophagy/lipophagy in the liver [61]. It was found that ATGL promotes autophagic flux in a SIRT1-dependent manner and promotes interactions between LC3 and lipid droplets (Figure 5). Furthermore, inhibition of autophagy suppressed ATGL-mediated lipid droplet breakdown and FA oxidation, suggesting that ATGL activates lipophagy. On the contrary, in colon epithelial cells, ATGL deficiency induces autophagic flux, whereas its co-factor ABHD5 promotes autophagy but in an ATGL-independent manner [276]. It was found that ABHD5 binds to and prevents the cleavage of the essential autophagy regulator Beclin 1 (BECN1), thus stimulating autophagy, which reduces colon cancer tumorigenesis [276]. Another enzyme from the PNPLA family has been recently shown to stimulate lipophagy [277]. PNPLA8, also named calcium-independent phospholipase A_2_ γ (iPLA_2_γ), displays PLA_2_ activity and preferentially hydrolyses arachidonic acid-containing membrane phospholipids [278]. PNPLA8 plays an important role in mitochondrial lipid and energy metabolism and its transgenic expression in the heart leads to TAG accumulation and cardiac dysfunction [196,197,279]. A recent study has shown that PNPLA8 interacts with autophagosomes and stimulates hepatic lipid droplet breakdown via autophagy in a mouse model of non-alcoholic fatty liver disease [277]. These studies add to the notion that an intricate relationship exists between lipid droplet lipolysis and autophagy/lipophagy and that lipases and their co-factors may act as important upstream regulators of autophagy and lipophagy [83]. 

### 8.3. Autophagy Drives Lipid Droplet Biogenesis to Protect Cells from Stress

As discussed above, the importance of autophagy for lipid droplet breakdown has been well established in the last decade, but autophagy also affects lipid droplet biogenesis. Studies in 3T3-L1 preadipocytes have shown that the depletion of Atg5 and Atg7 leads to a reduced lipid droplet accumulation and impaired differentiation [33]. Similar results were obtained in Atg5-depleted primary mouse fibroblasts, which failed to differentiate into mature adipocytes [280]. Accordingly, in vivo experiments have revealed that adipose-specific deletion of Atg7 significantly reduces adiposity, causes a shift towards a brown adipose tissue phenotype, including an increase in mitochondrial biogenesis and rates of FA oxidation, leading to an enhanced insulin sensitivity and a lean phenotype [33]. Furthermore, targeted deletion of Atg5 in late-stage embryos and neonatal pups reduced subcutaneous fat [280]. These findings illustrate that autophagy is required for normal adipocyte differentiation and the maintenance of lipid storage in adipose tissue in vivo but do not directly implicate autophagy in the mechanism of lipid droplet biogenesis. Two studies by Shibata et al. [241,281] have reported that autophagy is involved in lipid droplet synthesis in the liver, heart, and in various cultured cell lines. Atg7 deficiency led to a reduction in lipid droplet biogenesis in mouse hepatocytes and a co-localization of conjugated LC3-II with lipid droplets was observed in hepatic and cardiac tissues of starved mice [241]. In addition, depletion of LC3 resulted in a reduced lipid droplet accumulation in several cell types, including the cancer cell lines HeLa, HepG2, and PC12 [281], suggesting that the LC3 conjugation system is involved in lipid droplet formation. These studies suggested that the autophagic machinery regulates both autophagosome and lipid droplet biogenesis as a coordinated mechanism for the maintenance of cellular homeostasis. Recently, it was demonstrated that a liver-specific knockout of Atg5 leads to impaired fasting-induced lipid droplet biogenesis, suggesting that autophagy is involved in lipid droplet biogenesis from exogenous lipids provided from the circulation during fasting [242]. Interestingly, lipid droplet biogenesis was restored in Atg5/nuclear factor-like 2 (Nrf2) double-knockout mice, suggesting that persistent Nrf2 activation is responsible for the impaired lipid droplet biogenesis in autophagy-deficient livers. Nrf2 is a stress-activated transcription factor that regulates the expression of a range of antioxidant and stress-responsive genes [282]. This suggests that autophagy-dependent lipid droplet biogenesis in the liver is involved in the maintenance of redox homeostasis and protection from oxidative stress during fasting. 

The first clues for understanding the reasons for lipid droplet biogenesis in starved cells came from recent studies showing that autophagy contributes to lipid droplet biogenesis in acutely starved MEFs [25,31]. Genetic depletion of Atg5, inhibition of autophagosome formation by 3-MA and of autophagosome-lysosome fusion by bafilomycin A1, all prevented lipid droplet biogenesis during starvation in HBBS. Using a fluorescently-tagged phosphatidylcholine, Rambold et al. also show that autophagosomal degradation of membrane phospholipids provides FAs for lipid droplet synthesis. Their results further suggest that the role of autophagy in acutely starved cells is a constant replenishment of lipid droplets with FAs, which are then released by ATGL-mediated lipolysis and transferred to mitochondria for energy production. In the same model system, Nguyen et al. [31], have recently discovered that the function of autophagy-dependent lipid droplet biogenesis in starved MEFs is also to reduce the lipotoxicity of FAs released by autophagy. They show that lipid droplets serve as a protective buffer system that prevents mitochondrial damage and allows for a controlled lipolytic release of FAs for mitochondrial energy production. Importantly, lipid droplet biogenesis in starved MEFs occurs specifically in the absence of amino acids, but not during glucose or serum starvation, and is controlled by the major nutrient sensor and cell growth-associated kinase mTORC1 [31]. mTORC1 is active in the presence of sufficient amino acids to promote anabolic growth and is a potent negative regulator of autophagy [283]. Inhibition of mTORc1 in the presence of nutrients led to activation of autophagy and was sufficient to induce lipid droplet biogenesis, suggesting that autophagy-derived lipid droplet synthesis is not limited to starvation, but it may be important for various conditions characterized by high autophagic flux, including cancer [31]. Furthermore, the authors suggest that since mTORc1 responds to various nutrient and stress signals, mTORc1-dependent autophagy could explain the increase in lipid droplet accumulation under other stress conditions, such as ER stress [23,284], proteasome inhibition [285,286], and hypoxia/oxidative stress [8,24,28,204]. Indeed, several studies suggest that mTOR-dependent autophagy is activated during hypoxia [287,288], which opens the possibility that autophagy contributes to lipid droplet biogenesis during hypoxia and other conditions of oxidative stress. 

Autophagy-induced lipid droplet biogenesis has been recently associated with the protection of mesenchymal stem cells against nutrient and oxidative stress [34]. Carnitine palmitoyltransferase 1C (CPT1C) is an isoform of CPT1A with minimal catalytic activity and is expressed in the brain and many cancer cells [289,290]. In human mesenchymal stem cells exposed to glucose and oxygen deficiencies, CPT1C promoted autophagy-dependent lipid droplet biogenesis rather than directly affecting FA oxidation [34]. Inhibition of ATGL blocked the pro-survival effect of CPT1C and reduced ATP production, suggesting that ATGL-mediated lipolysis and energy production are required for its pro-survival effect [34]. In cancer cells, CPT1C is induced by hypoxia and glucose deprivation, its expression inversely correlates with mTORc1 activation and it promotes cell survival by stimulating FA oxidation and ATP production [290]. It is thus possible that CPT1C activates autophagy and lipid droplet biogenesis to promote cancer cell survival through lipid droplet lipolysis, which, in turn, provides FAs for oxidation in mitochondria and stimulation of PPARα-mediated mitochondrial biogenesis and oxidative metabolism [67]. Additionally, the link between autophagy and lipid droplet biogenesis during stress has been observed in other organisms. Rapamycin-induced autophagy contributes to TAG synthesis in yeast [291] and autophagy is required for TAG accumulation under nitrogen-deprived conditions in *Chlamydomonas* [292]. Clearly, autophagy-driven lipid droplet biogenesis is beneficial for a variety of cells in different stress conditions, but it remains to be confirmed whether lipid droplet biogenesis is indeed a general protective response to high levels of autophagy. The tight bonds between autophagy, lipid droplet metabolism, and the cellular stress response are only beginning to emerge and future studies will surely provide important clues on the molecular mechanisms involved and their pathophysiological relevance. This will help us improve our understanding of the specific alterations of lipid metabolism in cancer cells and their metabolic advantage during stress. 

## 9. Conclusions and Perspectives

Lipid droplets have long been regarded as inert fat storage depots and we know very little about their lipid and protein composition, dynamics, and functions in the cell. Along with the increasing interest in lipid metabolism in cancer in the last decade, there is an emerging appeal for studying lipid droplets and their role in the cellular stress response. The extra- and intracellular sources of lipids used by cells for lipid droplet biogenesis and the reasons behind lipid droplet accumulation in different pathophysiological settings are only beginning to emerge. It is also not clear how different stress conditions affect the choice of lipids for lipid droplet biogenesis and how lipid droplets regulate the release of particular species of lipids from their core. Likewise, one of the outstanding questions in cancer research is which lipid sources are used by cancer cells for survival in particular stress conditions and how cells switch between them. Furthermore, it will be important to get a broader and quantifiable picture of the lipid species stored in lipid droplets in various cells and tissues and improve our ability to follow the dynamic changes in the lipid droplet lipidome and proteome. For example, it will be fascinating to discover how different stress conditions and diseases are reflected by changes in the lipidome/proteome and what the roles of the numerous fatty acid, cholesterol and ceramide species stored in lipid droplets are. The potential of lipid droplets as lipid mediator signalling hubs is also emerging and provides exciting prospects for their role in inflammation and immunity. Identification of lipid droplet-associated proteins involved in sensing and responding to nutrient and oxidative stress will improve our understanding of lipid droplet functions in the stressed cancer cell. Studies will also provide important clues on the identity of lipid droplet proteins that are specifically recognized by the lipophagic machinery. It will also be important to see how cells switch between lipolysis and lipophagy and how these complementary processes of lipid droplet breakdown are regulated. Moreover, lipid droplet biogenesis may be a general cellular response to conditions of high autophagic flux. Clearly, we are only at the beginning of our understanding of the complex network of interactions between lipid droplets and the various forms of autophagy. Finally, interfering with lipid droplet biogenesis and/or breakdown may be beneficial for reducing the ability of cancer cells to cope with stress in therapeutic settings. The inhibition of lipid droplet biogenesis could compromise lipid acquisition and usage pathways in cancer cells by concomitantly increasing lipotoxicity and shutting down critical survival mechanisms. Current studies imply that several molecular targets, such as DGAT1, ATGL and LPCAT2, hold promise for reducing the availability of lipids critical for the survival, growth, and metastatic potential of cancer cells. However, given the pleiotropic roles of lipid droplets, it will be critical to first define the dependence of particular cancer types on lipid droplet metabolism. For example, targeting lipid droplet lipolysis (e.g., via inhibition of adipose tissue ATGL) may be particularly useful for the treatment of cancers that depend on and strive in the vicinity of adipose tissue, such as breast and ovarian cancer. ATGL inhibitors are currently under investigation as promising therapeutic agents for the treatment of metabolic disorders, but they may also prove to be beneficial in the prevention of cancers associated with obesity and diabetes. Future studies of the roles of lipid droplets in challenged (cancer) cells will surely provide important answers to many of these questions and will improve our ability to target the specific vulnerabilities of different cancer cells in their respective environments.
